# A Rare SPRY4 Gene Mutation Is Associated With Anosmia and Adult-Onset Isolated Hypogonadotropic Hypogonadism

**DOI:** 10.3389/fendo.2019.00781

**Published:** 2019-11-12

**Authors:** Rita Indirli, Biagio Cangiano, Eriselda Profka, Giovanna Mantovani, Luca Persani, Maura Arosio, Marco Bonomi, Emanuele Ferrante

**Affiliations:** ^1^Department of Clinical Sciences and Community Health, University of Milan, Milan, Italy; ^2^Endocrinology Unit, Fondazione IRCCS Ca' Granda Ospedale Maggiore Policlinico, Milan, Italy; ^3^Lab of Endocrine and Metabolic Research, Division of Endocrine and Metabolic Diseases, IRCCS Istituto Auxologico Italiano, Milan, Italy

**Keywords:** Kallmann syndrome, anosmia, isolated hypogonadotropic hypogonadism, central hypogonadism, SPRY4

## Abstract

**Background:** Isolated hypogonadotropic hypogonadism (IHH) is a rare, clinically heterogeneous condition, caused by the deficient secretion or action of gonadotropin releasing hormone (GnRH). It can manifest with absent or incomplete sexual maturation, or as infertility at adult-age; in a half of cases, IHH is associated with hypo/anosmia (Kallmann syndrome). Although a growing number of genes are being related to this disease, genetic mutations are currently found only in 40% of IHH patients.

**Case description:** Severe congenital hyposmia was diagnosed in a 25-year-old Caucasian man referred to the Ear-Nose-Throat department of our clinic. The patient had no cryptorchidism or micropenis and experienced a physiological puberty; past medical history and physical examination were unremarkable. Olfactory structures appeared hypoplasic, while hypothalamus, pituitary gland, and stalk were normal on MRI (neuroradiological imaging); testosterone levels, as well as pulsatile gonadotropin secretion and other pituitary hormones were unaffected at the time of first referral. At the age of 48, the patient returned to our clinic for sexual complaints, and the finding of low testosterone levels (6.8 and 5.8 nmol/L on two consecutive assessments) with inappropriately normal gonadotropin levels led to the diagnosis of hypogonadotropic hypogonadism. GnRH test was consistent with hypothalamic origin of the defect. Next generation sequencing was then performed revealing a rare heterozygous allelic variant in *SPRY4* gene (c.158G>A, p.R53Q). The biological and clinical effects of this gene variant had never been reported before. A diagnosis of Kallmann syndrome was finally established, and the patient was started on testosterone replacement therapy.

**Conclusion:** This case describes the clinical phenotype associated with a rare *SPRY4* gene allelic variant, consisting in congenital severe smell defect and adult-onset IHH; in patients with apparently isolated congenital anosmia genetic analysis can be valuable to guide follow up, since IHH can manifest later in adulthood. Characterization of other modifying genes and acquired environmental factors is needed for a better understanding of the physiopathology and clinical manifestations of this disease.

## Background

Isolated hypogonadotropic hypogonadism (IHH) is a rare condition, with an incidence of 1:250,000 in females and 1:30,000 in males (1). It is caused by the deficient secretion or action of gonadotropin releasing hormone (GnRH), and generally manifests as incapacity to start or complete sexual maturation at pubertal age, and infertility (2, 3). However, the syndrome is clinically heterogeneous as micropenis and cryptorchidism can be seen as early signs of severe GnRH deficiency occurring *in utero* in a male infant (4); on the other hand, some patients manifest hypogonadism only in adult life, after completion of pubertal development (*adult-onset IHH*) (2, 3, 5–7).

IHH results from a failure in GnRH neurons' development or migration, or from a defective GnRH secretion or action. During embryogenesis, indeed, immature GnRH neurons originate in the olfactory epithelium, and then migrate in close association with growing axons of olfactory nerves to finally reach the forebrain (8, 9). This shared route explains the association of IHH with defective olfaction in Kallmann syndrome (KS), which is found in about 50% of IHH cases (2); furthermore, other manifestations can be present in these patients, including synkinesis, dental agenesis, digital bony abnormalities, cleft palate, hearing loss, and unilateral renal agenesis (2, 5, 10). Despite most cases of IHH are sporadic, families with congenital IHH have been reported with X-linked, autosomal dominant and autosomal recessive inheritance (11), and several genes have been identified so far (10). Among these, some are mainly related to the disruption of GnRH neurons' development and migration (e.g., *ANOS1, FGF8, FGF17, FGFR1, IL17RD, DUSP6, SPRY4, GLCE, FLT3, PROK2, PROKR2, NSMF, WDR11, HS6ST1, SEMA3A, SEMA3E, CHD7, TUBB3, SOX10*), while others are implied in the neuroendocrinological regulation of GnRH secretion (*GNRH, GNRHR, KISS1R, KISS1, TAC3, TACR3, LEP, LEPR*) (12, 13). Genetics of KS and normosmic IHH are largely overlapping, but appear to explain only 40% of IHH patients, with other cases remaining genetically uncharacterized (14); a possibly more complex background is being disclosed in recent years, and oligogenicity and gene-environment interactions are emerging in the physiopathology of this disease (6, 15).

Here we describe the case of a male patient with congenital anosmia and adult-onset IHH, in whom a rare allelic variant in *SPRY4* gene was found. The biological and clinical significance of this mutation had never been reported before.

## Case Description

**Figure 1:**
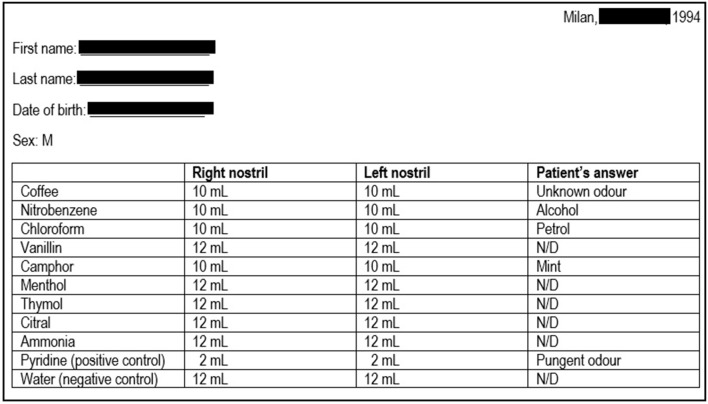
Results of the sniff test performed at patient's first referral. The first column indicates the 10 odorants employed; the second and the third columns indicate the minimum amount of each substance that the patient was able to detect on the right or left nostril, respectively (first threshold); in normal individuals, the first threshold is generally <3 mL. The patient was also asked to identify the substances, and the patient's answers are reported in the last column. The results are consistent with a severe smell defect. N/D, not detected.

In 1994, a 25-year-old Caucasian man presented to the Ear-Nose-Throat Department of our clinic for hyposmia and hypogeusia, which the patient stated to be present since infancy; a Standard Sniff Test was carried out, which was consistent with a severe smell defect (Figure 1). Considering the possible association with hypothalamus-pituitary-testis axis defects, the patient was then referred to the Endocrinology department. The patient had no cryptorchidism or micropenis at birth and had gone through physiological puberty, with complete development of primary and secondary sexual characteristics (Tanner stage 5); physical examination was unremarkable: he was 186 cm tall and weighed 75 kg (body mass index, BMI: 21.7 kg/m^2^), with normal arm span to height ratio and absence of gynecomastia; bitesticular volume was 35 mL, and he did not complain of any sexual symptom. Past medical history was significant only for surgical detorsion of the left testicle at the age of 13. No family history of infertility, hyposmia, or pituitary diseases was reported, and his parents were not consanguineous.

**Figure 2:**
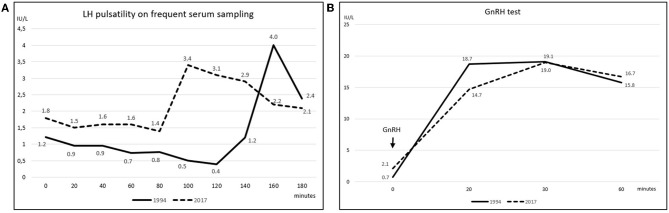
**(A)** Spontaneous LH pulsatility assessed by frequent serum sampling. The graph depicts LH pulsatility assessed at the patient's first referral in 1994 (solid line) and upon hypogonadism symptoms' manifestation in 2017 (dashed line) (LH reference values 1.7–8.6 IU/L); gonadotropin's levels were measured at 20-min intervals for 3 consecutive hours; secretion still occurred in a pulsatile fashion at both time points; nevertheless, as far as one single LH peak was evaluated, the LH release profile appeared slightly flatter in 2017 compared to the 1994 assessment. **(B)** LH response to GnRH stimulation test. GnRH stimulation test was performed in 1994 (solid line) and then repeated in 2017 (dashed line). Blood was drawn before, and then 20, 30, and 60 min after intravenous GnRH administration; a ≥400% increase from baseline was considered normal (16). LH curves were similar at the two time points and both responses resulted normal, as levels increased by 26 folds in 1994 and by 8 folds in 2017.

Early-morning total testosterone (TT) was 20.3 nmol/L (5.8 ng/mL; normal values, n.v., 12.0–29.1 nmol/L); basal Luteinizing Hormone (LH) and Follicle Stimulating Hormone (FSH) levels were 1.22 IU/L (n.v. 1.7–8.6) and 1.63 IU/L (n.v. 1.5–12.4), respectively, and they showed normal pulsatility on frequent serum sampling and normal response to GnRH test (Figures 2A,B); the remaining basal pituitary function was unaffected, as well (Table 1). Skull magnetic resonance imaging with contrast enhancement revealed absence of olfactory bulbs and tracts and hypoplasia of olfactory sulci, while hypothalamus, pituitary gland, and stalk were normal.

**Table 1:** Basal pituitary hormones' assessment in 1994 and in 2017.

	**Reference values**	**1994**	**2017**
TSH (mIU/L)	0.28–4.30	0.9	1.37
fT4 (ng/L)	8.0–17.0	16.5	11.2
Prolactin (mcg/L)	1.7–16.0	5.5	4.3
Cortisol (mcg/dL)	4.8–19.5	14.1	14.6
ACTH (ng/L)	0.0–46.0	N/A	24.2
IGF-1 (mcg/L)	50–200	N/A	148
Total testosterone (nmol/L)	12.0–29.1	20.3	6.8 and 5.8
LH (IU/L)	1.7–8.6	1.22	2.1
FSH (IU/L)	1.5–12.4	1.63	4.2

*TSH, thyroid stimulating hormone; fT4, free thyroxine; ACTH, adrenocorticotropic hormone; IGF-1, insulin-like growth factor 1 or somatomedin C; LH, luteinizing hormone; FSH, follicle stimulating hormone; N/A, not assessed*.

The patient was then lost to follow up until 2017 when, at the age of 48, loss of libido, reduction in spontaneous erections, and erectile dysfunction appeared. Bitesticular volume was 30 mL at this time, and a consistent weight gain occurred (BMI 25.9 kg/m^2^). Hormonal assessment showed definitely low testosterone confirmed on a second sampling (TT 6.8 and 5.8 nmol/L on two consecutive assessments, n.v. 12.0–29.1 nmol/L; calculated free testosterone 61 pg/mL, n.v. > 65 pg/mL). Gonadotropin levels were inappropriately-low (LH 2.1 IU/L, n.v. 1.7–8.6, and FSH 4.2 IU/L, n.v. 1.5–12.4), but with preserved response to GnRH stimulus (Figure 2B); LH secretion still occurred in a pulsatile fashion as assessed on frequent serum sampling; nevertheless, as far as one single LH peak was evaluated, LH release profile appeared slightly flatter compared to the 1994 assessment (Figure 2A). The findings were thus consistent with central hypogonadism (17) resulting from a hypothalamic defect. Ferritin levels were within the normal range. Other pituitary hormones were normal (Table 1), as well as number, motility, and morphology of spermatozoa in semen analysis.

Bone mineral density was preserved at lumbar spine (*T*-score −0.04, *z*-score −0.1), femoral neck (*T*-score +0.9, *z*-score +1.6), and total hip (*T*-score +1.0, *z*-score +1.3).

Genetic analysis on 28 loci was obtained by next generation sequencing (NGS). A single heterozygous missense allelic variant in the exon 3 of *SPRY4* gene (c.158G>A) was identified. This is a rare gene variant (minor allele frequency < 0.01) leading to an amino acid substitution at position 53 (p.R53Q), whose biological and clinical effects had not been known so far. No other variants in the 27 remaining loci were identified by NGS.

Following this result, a diagnosis of KS was finally established. Other anomalies that have been found to occur in KS, namely cleft lip and palate, synkinesis, sensorineural deafness, cerebellar ataxia, renal agenesis, digital bony abnormalities, or dental agenesis, were not present in the patient and his family.

The patient was not planning fertility and was started on replacement therapy with transdermal testosterone, which was effective in normalizing circulating testosterone levels and in improving sexual symptoms.

## Laboratory Investigations and Diagnostic Tests

Olfactory evaluation was performed by Standard Sniff-Test; this measure involves the unirhinal presentation of 10 odorants (including pure olfactory, olfactory-gustatory, and olfactory-trigeminal odorants) to each nostril for a total of 20 exposures. Pyridine and water were, respectively, used as positive and negative control. The test is based on the presentation to the patient of progressively increasing volumes (from 1 up to 12 mL) of air saturated with the specific olfactory substance, which must be first detected (first threshold; normal <3 mL) and therefore recognized (second threshold); the different thresholds are recorded, along with any wrong substance identification (Figure 1).

Frequent serum sampling for LH and FSH pulsatility was carried out by measurement of LH and FSH levels at 20-min intervals for 3 consecutive hours.

For GnRH stimulation test, intravenous GnRH 100 mcg was administered at 2-h intervals for three times when GnRH test was performed for the first time in 1994; blood samples for LH and FSH assessment were drawn before, and then 20, 30, 60, 90, and 120 min after GnRH injection. In 2017, a single bolus of GnRH was administered and blood samples for LH and FSH were collected at 0, 20, 30, and 60 min. A ≥400% increase from baseline was considered normal (16).

Basal circulating levels of cortisol, ACTH, prolactin, IGF-1, TSH, and fT4 were obtained for the study of the other hypothalamus-pituitary axes.

Several candidate genes for IHH were analyzed by Next Generation Sequencing. We extracted the genomic DNA of the patient from peripheral blood lymphocytes using Gene Catcher gDNA 96 × 10 mL Automated Blood kit (Invitrogen, Life Technologies™, City, Country). The IHH gene panel was designed using Illumina Design Studio (San Diego, CA, USA) and included the following IHH candidate genes: *ANOS1(KAL1), FGFR1, PROKR2, PROK2, GNRHR, GNRH1, GNRH2, KISS1, KISS1R, TAC3, TACR3, HS6ST1, FGF8, CHD7, DUSP6, FEZF1, FGF17, FLTR3, IL17, SEMA3A, SEMA3E, SEMA7A, SOX2, SOX10, SPRY4, WDR11, HESX1, NSMF (NELF)*. The 28 IHH genes consistently represented in all sequence capture panels were assessed for the purposes of this study. Libraries were prepared using Illumina Nextera Rapid Capture Custom Enrichment kits according to the manufacturer's protocols. All regions not correctly sequenced were recovered with NexteraVR DNA Library Preparation kit (Illumina, San Diego, CA, USA). We included as “rare variants” (Manolio, T.A, Nature 2009) all known pathogenic, or rare non-synonymous or splicing-site variants (Minor Allele Frequency, MAF ≤ 0.01) and novel non-synonymous or splicing-site variants. The frequency and the functional annotation of the identified variants were checked in public and licensed databases (Ensembl, UCSC Genome browser, 1000 Genome project, ExAC Browser, NCBI, HGMD professional), considering the ethnic group (Europeans). As previously reported (18), we excluded common non-synonymous variants with Minor Allele Frequency (MAF) >0.01, synonymous, intronic, and 5′ or 3′ UTR variants. Each variant found was confirmed by Sanger direct sequencing using BigDyeVR Terminator v.3.1 Cycle Sequencing Kit (Life Technologies, Carlsbad, CA, USA) on a 3100 DNA Analyzer from Applied Biosystems (Foster City, CA, USA).

## Discussion

IHH is a rare and clinically heterogeneous condition; a number of genetic alterations and non-genetic environmental triggers are being unraveled in recent years (5). In the present case report we had the unique opportunity to follow the natural clinical history of a subject that was originally affected with isolated congenital anosmia which evolved in a more complex phenotype in the adulthood. Genetic phenotyping demonstrated the presence of a rare allelic variant in *SPRY4* gene, whose biological and clinical significance has not been investigated so far^1^.

Indeed, the patient reported severe congenital hyposmia and adult-onset IHH, which became evident many years after completion of physiological puberty and was preceded by significant weight gain; olfactory structures were absent/hypoplasic on neuroradiological imaging. The hormonal assessment was indicative of a hypothalamic defect in GnRH secretion, while the remaining hypothalamus-pituitary axes were unaffected. This case appears to fit well with the adult-onset KS Italian cohort recently reported by Bonomi et al. (5) and showing a high prevalence of overweight, and a hormonal profile similar to the present case. Consistently, in the adult-onset form, weight gain may affect GnRH activity and LH/FSH pulsatile secretion, and may act as an acquired factor contributing to the onset of IHH in genetically predisposed patients (6, 19).

*SPRY4* (Sprouty RTK Signaling Antagonist 4) gene is located on chromosome 5 and encodes a member of the human/mouse SPRY family (SPRY1-SPRY4) (20). Particularly, *SPRY4* belongs to the so-called *FGF8 synexpression group*, a cluster of genes which not only share an expression pattern similar to that of *FGF8* during embryonic development, but also act by modulating *FGF8-FGFR1* intracellular signaling (21). The SPRY4 protein is an inhibitor of the RAS-MAPK pathway, since it impairs the formation of active GTP-RAS (20); it has been found to be expressed in the olfactory placode in mouse embryos, and in the hypothalamus of adult mice (22). *SPRY4* knockout phenotype includes craniofacial defects and abnormal limb development (23). Miraoui et al. have identified *SPRY4* allelic variants in 14 IHH patients, with both monogenic (*n* = 11) and oligogenic (*n* = 3) patterns of inheritance (24); in all patients hypogonadism had manifested before completion of physiological puberty; both nIHH and KS were observed. With regard to Miraoui's series, the mutation found in our patient manifests with a different phenotype, considering the late onset of hypogonadotropic hypogonadism and the congenital severe smell defect. On the other hand, as recently reported in another study evaluating both classic forms of IHH and adult onset forms (6), it seems that, to some extent, the same genes are implied also in mild diseases. In fact, *SPRY4* was reported to be one of the loci enriched in rare variants among patients harboring mild and adult onset IHH, compared with both controls and classic forms of hypogonadotropic hypogonadism. The case we are reporting perfectly fits those epidemiological data: even if the damaging potential of this new variant has to be clarified with functional analyses and co-segregation studies, the congenital severe anosmia and the olfactory structures' alterations increase the chances of causality, as these are known targets of this gene. Moreover, we can hypothesize that the *SPRY4* allelic variant, found in our patient, might be sufficient to determine an early impairment of the olfactory migration, while causing a fragility of the GnRH neuron functionality that become evident only later in the adult life. This may result from the degree of disruption of the SPRY4 protein function itself; alternatively, other unknown modifying genes, as well as *in utero* or acquired environmental factors may have impacted the IHH phenotype. Four patients with *SPRY4* allelic variant have been previously reported to manifest other non-reproductive features, namely hearing loss and abnormal dentition (24); these can be present in patients carrying *FGF8-FGFR1* pathway defects (12), but were not observed in the presented case.

In conclusion, we have reported for the first time the temporal effect of a rare *SPRY4* gene allelic variant found to be associated with congenital severe smell defect and adult-onset IHH, in the absence of other non-reproductive features; in patients with congenital anosmia genetic analysis can be valuable to guide follow up, since hypogonadotropic hypogonadism can manifest later in life. Characterization of other modifying genes and environmental factors influencing IHH phenotype is needed for a better understanding of the physiopathology and clinical manifestations of this disease.

## Data Availability Statement

The datasets analyzed for this study can be found in the GenBank repository (Accession MN556092.1) (https://www.ncbi.nlm.nih.gov/nuccore/mn556092).

## Ethics Statement

Ethical review and approval was not required for the study on human participants in accordance with the local legislation and institutional requirements. The patients/participants provided their written informed consent to participate in this study.

## Patient Consent

Written informed consent was obtained from the patient for the publication of this case report.

## Author Contributions

EP, EF, and MB performed patient follow-up, clinical diagnosis, and treatment. MB, BC, and LP were responsible for genetic analysis and its interpretation. RI and EF collected clinical data and prepared the manuscript. EF, MB, LP, GM, and MA performed the critical revision of the manuscript. All authors contributed to manuscript revision, read, and approved the submitted version.

### Conflict of Interest

The authors declare that the research was conducted in the absence of any commercial or financial relationships that could be construed as a potential conflict of interest.

## Footnotes

^1^https://www.ncbi.nlm.nih.gov/snp/rs759316507#clinical_significance
